# Hyperledger Fabric Blockchain for Securing the Edge Internet of Things

**DOI:** 10.3390/s21020359

**Published:** 2021-01-07

**Authors:** Houshyar Honar Pajooh, Mohammad Rashid, Fakhrul Alam, Serge Demidenko

**Affiliations:** 1 Department of Mechanical and Electrical Engineering, Massey University, Auckland 0632, New Zealand; m.a.rashid@massey.ac.nz (M.R.); f.alam@massey.ac.nz (F.A.); 2 School of Science and Technology, Sunway University, Subang Jaya 47500, Malaysia; sdemidenko@Sunway.edu.my

**Keywords:** Internet of Things, hyperledger fabric, smart contract, security and privacy, data provenance, edge computing

## Abstract

Providing security and privacy to the Internet of Things (IoT) networks while achieving it with minimum performance requirements is an open research challenge. Blockchain technology, as a distributed and decentralized ledger, is a potential solution to tackle the limitations of the current peer-to-peer IoT networks. This paper presents the development of an integrated IoT system implementing the permissioned blockchain Hyperledger Fabric (HLF) to secure the edge computing devices by employing a local authentication process. In addition, the proposed model provides traceability for the data generated by the IoT devices. The presented solution also addresses the IoT systems’ scalability challenges, the processing power and storage issues of the IoT edge devices in the blockchain network. A set of built-in queries is leveraged by smart-contracts technology to define the rules and conditions. The paper validates the performance of the proposed model with practical implementation by measuring performance metrics such as transaction throughput and latency, resource consumption, and network use. The results show that the proposed platform with the HLF implementation is promising for the security of resource-constrained IoT devices and is scalable for deployment in various IoT scenarios.

## 1. Introduction

Internet of Things (IoT) [1] technologies are associated with the significant growth of generated, collected and used data. At the same time, with the rapid involvement of distributed heterogeneous devices, various aspects of traditional IoT applications and platforms face challenges in security, privacy, data integrity, and robustness [2]. The blockchain has emerged as an innovative engine that can facilitate reliable and transparent data transactions. It has been widely applied to traditional sectors, including finance, commerce, industry, and logistics.

Most IoT platforms and applications depend on centralized architecture by connecting to cloud servers via gateways. Unfortunately, this leads to severe security and privacy risks. Wireless communication between sensor nodes and IoT gateways might also be very susceptible to attack. Cloud servers are potential targets for Distributed Denial-of-Service (DDoS) attacks resulting in significant infrastructure collapse [3]. Moreover, the centralized server solution introduces a single point of failure risk to the entire system.

Networked devices in an IoT system are heterogeneous in terms of their security requirements and resource availability. Resource-constrained devices operate in an open environment that increases the risks of physical and wireless accessibility by adversaries. RSA (Rivest–Shamir–Adleman) [4] and ECC (Elliptic Curve Cryptography) [5] are the two most popular key cryptosystems. However, computing RSA is time-consuming due to the modular exponentiation involved. Similarly, point multiplication in ECC relies on modular multiplication, which is computation-intensive thus resulting in a prolonged operation. The computational complexity of conventional security techniques such as SSL (Secure Sockets Layer) [6] and its successor, TLS (Transport Layer Security), make them not suitable for IoT devices. The SSL/TLS approach supported by CRL (Certificate Revocation List) creates scalability challenges for IoT applications. Homomorphic encryption [7] is very useful in protecting the privacy of users. However, the homomorphic encryption may be slow thus requiring special implementation techniques to speed up the execution. The ideal solution must provide data security and integrity while handling vast traffic and being attack-resistant. Furthermore, lightweight, scalable, transparent access control are to be associated with such a model. Blockchain is regarded as a promising solution to provide decentralized accountability and an immutable approach that can be used to overcome the aforementioned problems in heterogeneous scenarios [1]. It offers great security features while providing high transparency and enhancing efficiency. Meanwhile, it can also improve data traceability and eliminate third-party intervention at a lower cost.

Thanks to the development of edge computing platforms, data generated by the IoT devices can be transferred to the edge gateways for further process and analysis. At the same time, cloud-centric services are not suitable for the edge computing applications due to the limited network bandwidth, security, and data privacy. When applied to the edge computing systems, the blockchain provides a feasible solution to protect IoT data from being tampered [8]. It is a general distributed, decentralized, and peer-to-peer system that guarantees data integrity and consistency within existing industrial domains. Ethereum [9] is a common blockchain service showing intrinsic characteristics of distributed applications (dApps) over the blockchain network such as decentralization, anonymity, and auditability. However, common blockchain platforms (e.g., Ethereum) require tremendous computational power, making the integration of IoT nodes challenging.

The blockchain is an emerging technology playing a vital role in storing information and securing IoT systems and devices [10]. Although the blockchain is a promising application to solve IoT privacy and security challenges of current centralized systems, lots of IoT devices are constrained to perform complex operations due to their limited power of CPU, restricted data storage, and constrained battery resources. Furthermore, existing consensus algorithms in blockchain-based networks such as the Proof of Work (PoW) [11] cannot be implemented on devices with limited computing resources. The mining process described as taking decisions by all the nodes in peer-to-peer networks, requires considerable computational capabilities. Smart contracts present another promising application of blockchain technology that can distributively enforce various access control policies in IoT applications in the real-world scenarios. The data provenance plays a decisive role in the security and privacy of IoT systems. Additionally, the integrity of all generated data by IoT devices can be ensured by private blockchain technology.

In this paper, a blockchain-enabled edge computing approach is proposed and implemented for the IoT network with an open-source Hyperledger Fabric (HLF) blockchain platform. HLF is the best fit for this study because of its lower processing complexity (fewer number of transactions). Moreover, the transactions there can be performed in parallel while using various validators. Additionally, the processing is made more efficient by employing the fast RAFT [12] consensus algorithm. Finally, it provides a channel mechanism for private communication and private data exchange between members of a consortium. Moreover, all the HLF programs run in the docker [13] containers providing a sandbox environment that separates the application program from the physical resources and isolates the containers from each other to ensure the application’s security. A layer-wise security architecture is designed according to the capabilities of different nodes and functionality to fit the scalable IoT applications. The infrastructure includes Base Stations (BS), Cluster Heads (CH), and IoT devices facilitating access control policies and management. Mutual authentication and authorization schemes for IoT devices are proposed and implemented with the aim to ensure the security of the interconnected devices in the scalable IoT platform. The local authentication is used for ordinary IoT devices connected to CHs (edge IoT gateways), while the blockchain service provides the authentication of the IoT edge gateways i.e., the edge IoTs. The practical end-to-end lightweight HLF prototype for IoT applications is deployed on the embedded edge IoT hardware built upon the ARM64 CPU-based Raspberry Pi to validate the feasibility of the proposed design. HLF docker images are customized to fit with the IoT gateways. The Fabric client facilitates the request and query of transactions through invoking ChainCodes (CC) in IoT gateways. Off-chain data storage and blockchain distributed data storage are employed to support the architecture data traceability. HLF is implemented to act as a medium for multiple device interactions while exchanging information. Moreover, the blockchain maintains a global computation state. The distributed data storage is secure, and it has a large capacity. The data processing confidentiality and efficiency are guaranteed by implementing external off-chain computations. An HLF blockchain middle-ware module embedded in the IoT gateways ensures secure data transactions for the IoT distributed applications. The performance metrics such as throughput, latency, resource consumption and network use of the proposed model are evaluated using the edge IoT devices and x86-64 commodity virtual hardware.

The following distinct contributions are made in this work:

1. A novel architecture for the security and privacy of IoT edge computing using a permissioned blockchain is proposed. The proposed architecture considers 5G-enabled IoT technologies for node communications. The architecture is suitable for real-world IoT systems due to the developed ChainCodes that facilitate storage and retrieval of data in a tamper-proof blockchain system. Moreover, blockchain-based data traceability for 5G-enabled edge computing using the HLF is designed to provide auditability of the IoT metadata through a developed NodeJS client library.
2. The adaptability of the Hyperledger Fabric for ARM architecture of the edge IoT devices is improved by modifying official docker images from the source as there are no official or public images of HLF to support the 64-bit ARMv8 architecture.
3. A lightweight mutual authentication and authorization model is designed to facilitate a secure and privacy-preserving framework for IoT edge that protects the sensor nodes’ sensitive data through a permissioned fabric platform. Furthermore, it provides trust for the IoT sensors, edge nodes, and base stations by the private blockchain. This is achieved by using the edge nodes to record the IoT data in an immutable and verifiable ledger to guarantee metadata traceability and auditability.
4. Performance characteristics of the proposed architecture blockchain in terms of throughput, transaction latency, computational resources, network use, and communication costs are experimentally evaluated in two network setups.

The rest of the paper is organized as follows. In Section 2, a review of the related works is presented. Section 3 presents the main characteristics of blockchain technology. Section 4 describes the proposed HLF model implementation and elaborates on the details of the system design. In Section 5 the profiling and analysis are presented, including results from real-life IoT applications. Finally, Section 6 presents the conclusion and directions for future work.

## 2. Related Work

### 2.1. IoT Overview

In general terms, IoT is a collection of physical devices, computers, servers, and small objects embedded within a network system [14]. Some of the most prominent IoT application areas are smart homes [15] and smart cities [16], vehicular systems [17], and smart healthcare networks [18]. All these systems are highly distributed. The evolution from the conventional cloud-centric architecture has been accelerated by the emergence of the edge computing technologies [19,20]. A unified standard classification is defined to ensure the consistency of the development and structures of IoT. It includes four layers: service layer, platform layer, network layer, and device layer [21]. A comprehensive review of security attacks towards Wireless Sensor Networks (WSNs) and IoT is presented in [22]. The study also provides the techniques for prevention, detection, and mitigation of those attacks.

IoT systems normally include many interconnected IoT devices generating a massive amount of data. Meanwhile, IoT devices normally have limited capabilities in terms of the CPU processing performance, memory capacity, and battery energy volume. Therefore, they can be characterized as having restricted ability to resist various cyber-attacks. This leads to issues associated with insufficient security and potential compromising of privacy. New technologies have been developed to address the IoT’s decentralization challenges with the blockchain being among the most promising of them.

### 2.2. IoT Blockchain

Most IoT applications are prone to problems such as system failure and data leakage. Blockchain technology can mitigate these problems by providing better security and scalability for IoT applications. However, there are many challenges associated with the actual implementation of the approach. They are associated with tasks distribution between IoT devices as well as with the limited capabilities of the IoT devices such as computational performance, memory capacity, power resources. Numerous research works on blockchain technology focus on coping with these challenges to adopt blockchain in IoT [23,24,25].

Many distributed and decentralized IoT systems have adopted blockchain technology to provide trust [26], security [27], data management [28], fault-tolerance [29], as well as peer-to-peer and interoperable transactions [30]. The application scope of blockchain platforms can be divided into three main types depending on the way they manage user credentials: (i) public or permissionless blockchain, (ii) private or permissioned blockchain, and (iii) consortium blockchain. Blockchains that anonymous nodes can join, read data, and participate in transactions with equivalent status are public blockchains. In contrast, private or consortium blockchains are based on permissions and different types of nodes. Some nodes need to be authenticated to perform specific actions [31].

Scalability is the major challenge in the integration of blockchain and IoT systems. Many research works have addressed the scalability issues within Bitcoin’s architecture [32]. Smart contracts are promising solutions to facilitate the integration of distributed IoT systems and blockchain technology. However, their performance and scalability are directly linked to overall blockchain system performance [33]. Multiple IoT applications recently adopted blockchain for digital payment, smart contract services [34], and data storage [35]. Nonetheless, continuous developments have shown that new technologies can bring significantly higher scalability and degree of performance to next-generation blockchain systems.

The layer-based IoT blockchain frameworks are proposed in the literature to cope with the scalability challenges in IoT systems while providing higher performance and security. The layer-wised structure is a promising solution to smart cities’ security by integrating smart devices and blockchain technology [36]. A hybrid-network architecture is seen to leverage the strength of emerging Software Defined Network (SDN) and blockchain technologies in a multi-layer platform [37]. Layer-based blockchain can potentially address the IoT systems’ challenges such as response time and resource consumption [38]. This approach can further facilitate the integration of blockchain technology in IoT systems by tackling the complexity of blockchain implementation in the layer-based model [39].

Security challenges associated with the cyber-physical systems (CPSs) of smart cities are reviewed in [40] and adoption of distributed anomaly detection systems by CPSs of smart cities is proposed. A permissioned private blockchain-based solution in the context of the Industrial IoT (IIoT) is proposed in [41] to secure the encrypted image. This approach stores the cryptographic pixel values of an image on the blockchain, ensuring the image data privacy and security. The state of the art in industrial automation is presented in [42] to provide a better understanding of the enabling technologies, potential advantages and challenges of Industry 4.0 and IIoT. Also, it covers the cyber-security related needs of IIoT users and services.

### 2.3. Blockchain for Mobile Edge Computing

Several pieces of research have considered the integration of blockchain technology and edge computing layer over the past few years. Multiple works have focused on enabling secure and efficient distributed edge computing [43,44]. Such integration targets security enhancement. It also uses blockchain technology to develop access control policies for various applications at the edge [45,46,47]. Other works [48,49] investigated the edge resource management by implementing the blockchain. Distributed robotic system automation was also considered [50]. The integration of blockchain significantly benefits the security of edge computing [51]. Permission blockchain and Distributed Ledger Technology (DLT) embedded with identity management bring benefits to address many challenges by adding a resilience layer while network traffic integrity is guaranteed against malicious diversion and traffic manipulation. Network resource manipulation and fraudulent use of shared resources are avoidable through the blockchain-enabled resource management. Moreover, the blockchain provides a higher degree of security for the automotive sector [48] and the healthcare sector at the edge [52]. Blockchain is applied to provide a decentralized authentication model in edge and IoT environments [53]. The blockchain application is further explored to enhance the privacy, integrity, and authentication between IoT, mobile edge computing, and cloud in telehealth systems connected with 5G and IoT [54]. An HLF-based blockchain architecture is proposed in [55] for healthcare monitoring applications. The authors in [56] highlighted the importance and benefits of fog computing for IoT networks. The study also provides a comprehensive investigation of hardware security to fog devices through an enriched literature review. A model based on HLF blockchain is proposed in [57] as a service to answer IoT systems’ specific requirements, including low hardware, storage, and networking capabilities.

### 2.4. Blockchain for Data Sharing and Traceability

Digital signatures and Message Authentication Code (MAC) are two standard methods to identify data lineage and origin. However, these cryptographic techniques are not able to provide comprehensive data provenance [58]. Furthermore, the key management in a heterogeneous IoT network with data sourced from different nodes is complicated. Although logging-based methods can facilitate data transmission and system events monitoring, they cannot efficiently track data in distributed IoT systems [59]. Blockchain technology has been widely considered for data provenance within a distributed system such as IoT. Data operations are embedded in the blockchain transactions to provide the data provenance [60]. ProvChain [61] is a distributed and decentralized blockchain-based data provenance architecture to provide verifiability and data integrity in cloud environment. A blockchain network records the data operations as the provenance of data in the blockchain transactions while the system stores the data record in a local ledger. Smart contracts can automate the blockchain-enabled provenance systems without the off-chain verification [62]. A function for tracing the data deviation is designed into smart contracts with built-in access rules to protect data privacy in a distributed ledger [63]. SmartProvenance [64] is the blockchain-based distributed data provenance system that facilitates the verification of provenance records and provides trustworthy data and provenance collection using smart contracts and the Open Provenance Model (OPM). The blockchain is proposed to ensure secure and trustworthy industrial operations [65]. The complexity of blockchain implementation causes various limitations in deploying the aforementioned provenance techniques in IoT systems. Existing works on data provenance are computationally complex and pose a hardware cost. Therefore, these methods are not feasible for resource-constrained IoT systems with limited CPU performance, memory size, and power capacity.

Despite the benefits that blockchain brings to IoT applications, there are resource constraints and scalability challenges associated with the integration [2,66,67]. Generally, the blockchain demands substantial computational power for the mining process in Proof of Work (PoW), low latency, and high bandwidth. IoT devices with low processing power are not capable of performing the blockchain mining process. The data encryption process is frequently happening in blockchain systems. The computationally intensive process of blockchain drains the low power capacity of IoT devices. The size of the blockchain ledger increases continuously while the storage capacity of most IoT devices is low. Storing a copy of the full blockchain ledger for IoT devices is not feasible as it requires a large memory capacity. With Bitcoin, the blockchain storage size rests at over 200 GByte while for Etherum it is around 1.5 TByte. New block generation and agreement reaching in the blockchain require the nodes to exchange information through the consensus process frequently. The consensus process and information exchange need high bandwidth and low latency. However, the bandwidth of IoT devices is normally strictly limited.

One common concern about the blockchain system is associated with the need for achieving high scalability in a blockchain network [68]. The problem with such a large blockchain size is centralization risk. Most IoT systems have a very high number of interconnected devices. In addition, IoT networks frequently change to suit different applications by adding or removing IoT devices. Therefore, a solution is required to address the IoT system scalability challenges. Moreover, the limitations in the processing power and storage capacity of IoT devices in the blockchain network are also to be resolved. Addressing these challenges is the main focus of this paper.

## 3. Blockchain Overview

Satoshi Nakamoto, first implemented a decentralized digital currency in 2009 [69]. The blockchain can be described as a distributed ledger consisting of immutable and verifiable transactions. All network participants share a replica of the ledger in the network. Integrity, immutability, transparency, non-repudiation and equal rights are the main properties of the blockchain systems.

Bitcoin [70] is known as the most popular blockchain platform. PoW is used in Bitcoin to perform ownership management and tracking coins owner via implementing public-key cryptography with a consensus algorithm. The consensus algorithm is executed when a new block is introduced to the previous block to guarantee the reliability and validity of all transactions. The nodes will reach a consensus when 51% of the nodes are truthful.

IOTA [71] is a distributed ledger designed for IoT to facilitate the value and data exchange. A machine-to-machine communication is facilitated by the Tangle protocol capable of forming micro-payment systems. Additionally, it establishes IOTA network, which is a set of Tangle graphs. This set constitutes the ledger to store transactions submitted by the network nodes. The process of block validation leads to making a decision and adding a new block to the blockchain.

### 3.1. Consensus Algorithm

Li et al. [72] reviewed the most common consensus algorithms in the existing blockchain systems. These consensus mechanisms are PoW, Proof of Stake (PoS), Practical Byzantine Fault Tolerance (PBFT), Delegated Proof of Stake (DPoS), Proof of Authority (PoA), Proof of Elapsed Time (PoET), and Proof of Bandwidth (PoB).

PoW is the widest deployed consensus algorithm [73] that was first introduced by Bitcoin. The nodes use computational power to compete in finding the nonce value. This process is called mining. The difficulty level for PoW is adjustable when the number of participants increases to manage the block’s average processing time. Higher difficulty results in a lower number of blocks. No user should take more than 50% of the processing power to avoid controlling the system by just one user.

PoS [74] was introduced to address the vast energy consumption issues associated with the competing process in PoW. No competition is employed in the PoS algorithm. The network selects a node as a validator (so-called a transaction validator node). The node is chosen in advance to be a part of the Proof of Stake and attend a similar process of difficulty adjustment as PoW. If the validator does not validate the transaction, the network sets the next node as a validator, and the process continues until any node validates the transaction. PoS deploys CASPER protocol to perform the consensus process.

PoA [74] algorithm is based on a chosen set of trusted nodes (known as Authorities). This consensus algorithm is a Byzantine Fault Tolerant (BFT) variation. The chain becomes a part of the permanent records when most authority nodes (for example at least N/2 + 1) signs off the chain. This procedure facilitates the creation of a permissioned chain and is associated with a lighter exchange of messages.

**Figure 1:**
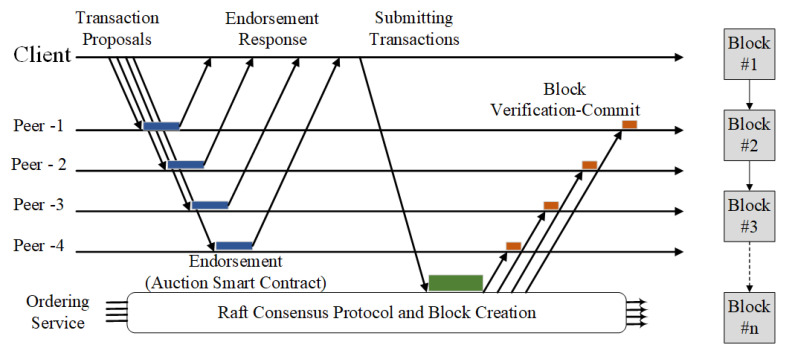
Overview of the RAFT consensus protocol and block creation.

Hyperledger [75], introduced in 2016 by the Linux Foundation, is the most successful and the most popular permissioned blockchain in the industrial and IoT domains. The designed permissioned blockchains for enterprise ecosystems deploy the RAFT Consensus Protocol [12], which is a better fit because it is more straightforward and less resource consuming. Figure 1 shows the process of the RAFT consensus protocol and block creation considered in this study.

Kafka [76] and RAFT are the same types of consensus that use Crash Fault Tolerant (CFT) for ordering service implementation. They can tolerate up to N/2 system failures. RAFT follows a “leader and follower” approach. There a leader node is dynamically elected among the ordering nodes in a channel (this collection of nodes is known as the “consenter set”), and the followers replicate its decisions. However, RAFT’s ordering service deployment is easier and more manageable than Kafka-based ordering services from the configuration to the process’s speed. Additionally, the RAFT configuration originates directly from the orderer (unlike the Kafka case, which cannot be configured directly from orderer services and must create a Zookeeper cluster to enable the state machine replication process). The comprehensive design facilitates different organizations to contribute nodes to a more distributed ordering service.

The process is initiated by sending the transaction proposals to the blockchain peers. A transaction proposal consists of various values, IoT metadata as well as other blockchain-related contents. The client application is responsible for starting the process and then transaction broadcasting to each blockchain member organizations’ peers. Once the peers receive the transactions, they activate the endorsement process by executing the ChainCode implementing authentication and authorization mechanism. The transaction is then endorsed and returned as the signed transaction. When all peers have endorsed the transaction based on the endorsement policy, the next step includes sending the transaction to the ordering service when the consensus is reached (i.e., RAFT in our case). The last step is encompassing the creation of the final block and committing it to the ledger.

### 3.2. Smart Contracts

Smart contracts are executable distributed programs to facilitate, execute, and enforce the terms of an agreement on a decentralized consensus tamper-proof and typically self-enforcing through automated execution [77]. The smart contracts are simply executable scripts that are filed on the blockchain with a specific address.

Smart contracts are triggered by transactions to execute and perform operations based on recorded instructions. They are installed and instantiated on blockchain participants. HLF is programmable by a construct called ChainCode (CC). Conceptually, CC is the same as the smart contract on other distributed ledger technologies. CC sits next to the ledger. Participants of the network can execute CC in the context of a transaction that is recorded in the ledger. Automation of business processes through CC leads to higher efficiency, transparency, and greater trust among the participants. Smart contracts allow decision automation thus making them suitable for IoT applications.

## 4. Hyperledger Fabric IoT System Model

### 4.1. Overall Design

**Figure 2:**
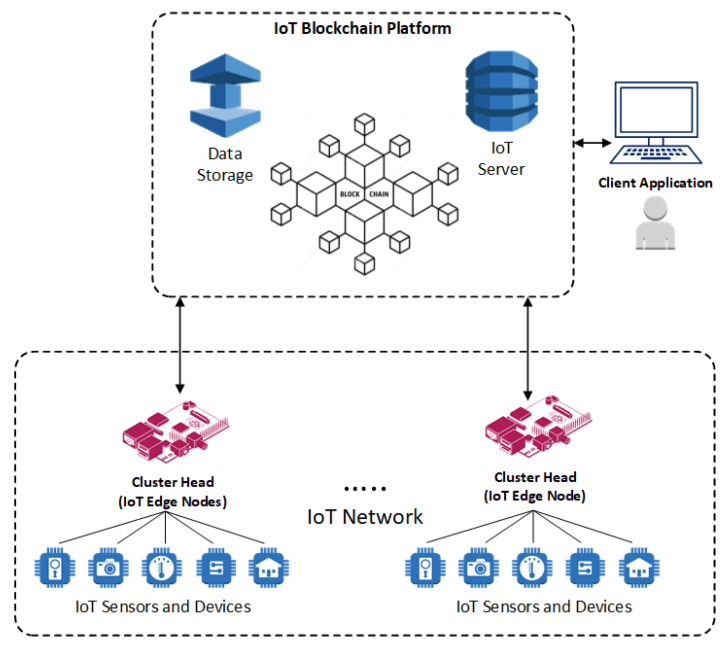
Conceptual framework of the integrated IoT blockchain platform.

The network model proposed in this work is based on blockchain technology as an individual application integrated with edge computing to provide security, identity management, and authentication. This study builds on the model introduced in our previous work [78] using a multi-layer platform approach and the Lightweight Hyperledger Blockchain technology along with smart contracts to enhance the performance of the blockchain-IoT combination. The whole network is divided into several layers and sub-networks. The devices in each layer have different computational capabilities and energy storage capacity. As a result, different security approaches are proposed for individual layers based on the blockchain. However, the blockchain implementation is modified to suit the devices of each particular layer. These layers are Base Station (BS) nodes, Cluster Head (CH) nodes (edge layer), and IoT devices. In the current work, we propose an additional layer—Off-Chain Storage servers—to enhance the data storage of IoT devices. Moreover, it facilitates the system performance improvement as the increase in the shared ledger size causes system performance degradation. The Hyperledger Blockchain platform is considered to be a potential solution to cope with scalability challenges while distributed programs are defined to facilitate various tasks and transactions [79]. However, the blockchain implemented in the embedded edge gateways provides reliable connectivity considering sufficient power and computational resources requirements. Figure 2 shows the conceptual framework of the proposed IoT Blockchain platform. The presented model encompasses interconnected IoT devices, Edge IoT nodes (CHs), client application nodes, external data storage, and IoT servers orchestrated in the peer-to-peer blockchain-based network to form a multi-layer blockchain model.

### 4.2. Multi-Layer IoT Blockchain Network

#### 4.2.1. Layer-1

A cluster of IoT devices is collected under each CH, a service agent for that cluster. This layer is the external service interface, in which IoT devices collect sensing data, perform local computing, and send results for storage and further analysis. CH nodes register the identity of each connected IoT device by implementing a smart contract. Each IoT device has a unique address within the IoT system. Each IoT node exists only in one cluster. The nodes in this layer have limited power, computational performance, and storage resources.

#### 4.2.2. Layer-2

Cluster heads at Layer-2 are responsible for data routing, security management (such as local authentication and authorization procedures), and network management. Beyond the aforementioned responsibilities, the IoT blockchain service is running in this layer to provide blockchain technology services and form a distributed system. The IoT devices’ identity management, communications, and consensus processes are run in this layer within the peer-to-peer blockchain network. The blockchain also handles the shared distributed ledger across all participants. Furthermore, this layer handles consensus algorithms and smart contract services to form data consistency and traceability.

A client application node across the network can have granted access to invoke various blockchain behaviors. Various ledger modifications are enabled by running smart contracts installed and instantiated in all peer nodes or selected peer nodes. The CH nodes running local authentication and authorization mechanism are directly connected to BS nodes. ChainCodes provide deployment, query, and invocation services. The API rest server can act as an interface by the client application with modifying the network-related operations and behaviors. Furthermore, the application client performs transaction submission to the blockchain. Therefore, various services can be defined within the blockchain network, including user enrollment and authentication and IoT device registrations. The IoT device authentication and authorization need to be carried on before transaction submission. The local authentication and authorization process manages this procedure. Consequently, a registered participant can sign a transaction using its private keys.

Data queries are enabled through CC, which is an executable logic hosted by peer nodes. Additionally, it facilitates appending data from data stored in the ledger. CC and related functionalities are mirrored across all peer nodes. CC deployment can be done to a specific number of peers to address the scalability issues. Therefore, parallel execution can be supported, which is resulted in an overall increase in system performance. The client application performs several operations, including storing the data checksum, data pointers, and data ownership in the blockchain. The actual data is stored in an external data storage, which is off-chain.

#### 4.2.3. Layer-3

In general, this layer is consistent with the current centralized cellular network encompassing Base Station nodes while the cloud server manages the process requests and data generated from various devices. Powerful devices in this layer can choose to use a non-symmetric encryption algorithm for data transmission. Layer-3 provides connectivity and wide area networking capabilities for the edge nodes. Network in the Layer-3 is decentralized, and BS units are distributed. The nodes trust the BSs in the system while they can access public networks.

#### 4.2.4. Layer-4

This layer is designed for storing sensed data by the IoT devices as well as enabling big data analytic applications for further analysis. It is generally done off-chain. It stores the actual data, while the blockchain ledger data includes data checksum, pointers, and data ownership. The blockchain world state is stored in a database such as LevelDB or CouchDB. The stored data in be queried and traced by a file ID in the blockchain. This method provides data provenance and data consistency between the edge nodes.

### 4.3. Local Authentication and Authorization of IoT Devices in Layer-1

Identity of IoT devices is registered and stored in the shared ledger. Each IoT device can join only one cluster. The registration request is sent to CH. It includes the required information such as IoT node ID, cluster identity, and timestamp. CH runs the smart contract in the local blockchain to perform the IoT device registration. The mutual authentication model is designed to provide the security of IoT devices with limited resources. The role of CH is to register the IoT devices as well as locally authenticate and authorize IoT entities. It also interacts with other cluster heads to form a secure communication between entities through the implemented blockchain network.

**Figure 3:**
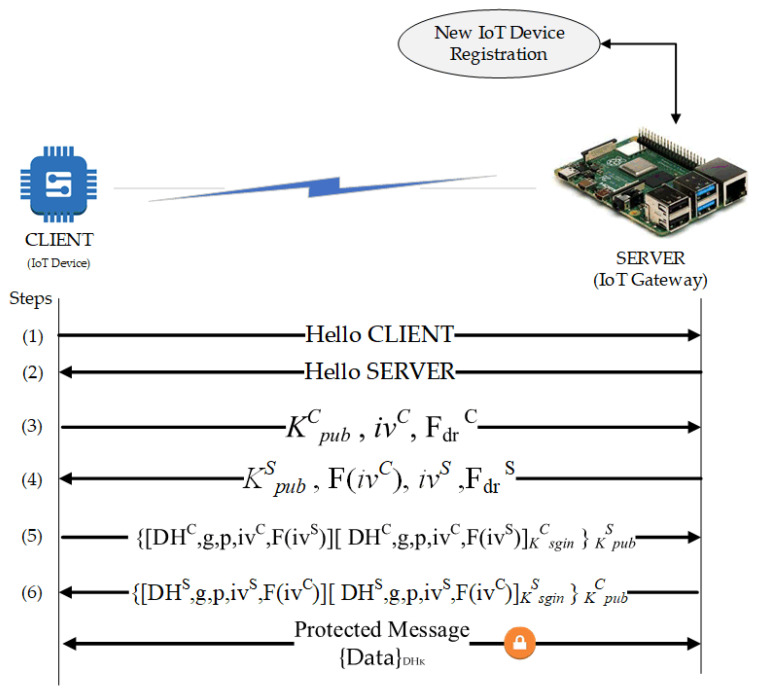
Local authentication flow.

The entire process is orchestrated in a smart contract to form an Authentication and Authorization ChainCode. The CC is installed and instantiated by the blockchain peer to perform the IoT blockchain local authentication procedure. This process is illustrated in Figure 3.

Authentication of the IoT devices consists of a few steps: the discovery of devices, key exchange, authentication, and data encryption. These procedures consider two network entities: the CLIENT (IoT sensor nodes) and SERVER (an edge computing gateway or intermediary node). It is noteworthy that the authentication of the IoT devices implements the exchange of keys using Diffie-Hellman Ephemeral (DHE) for the collection of session keys or secret keys. The following six steps describe the local Authentication of IoT devices.

Step 1 The first step starts with the CLIENT sending a package to the SERVER to establish a “connection”. For visualization purposes, this package contains the “HELLO CLIENT” character string.
Step 2 The answer from the SERVER to the CLIENT with the “HELLO SERVER” string. With that, the connection is established. For better performance, it is suggested to use chain bits for establishing the connection.
Step 3 The CLIENT generates a pair of asymmetric keys consisting of the public key (K^C_pub_^) and the private key (K^C_priv_^). For the key generation, an Initialization Vector (*IV*) is required with random values guaranteeing the distinction between the generated keys. Then, a packet is sent to the SERVER containing: the CLIENT’s public key (K^C_pub_^); a value such as “challenge-response” generated by the CLIENT; a character string F_dr_ defining the “challenge-response”.
Step 4 The SERVER generating a pair of asymmetric keys: the public key (K^S_pub_^) and the private key (K^S_priv_^). In sequence, the SERVER receives the CLIENT’s package and responds with another package containing its public key (K^S_pub_^) and the response to the “challenge-response” calculated from the F_dr_ function. The F_dr_ is a mathematically predefined function that can be sum, subtraction, or multiplication applied to the value of *IV* received.
Step 5 The CLIENT calculates Diffie-Hellman values. A new package consisting of the obtained DH value (*DH^C^*), the parameters *g* and *p* used in the calculation, a new value of *IV (iv^C^)*, and the value of *IV* obtained from the SERVER applied to the function F_dr_ *(F (iv^S^))* will be sent to the SERVER. Moreover, a summary function (Hash) for all these data and its result is encrypted with the CLIENT key (K^C_priv_^). It is then included in the package. The whole package is then encrypted with the public key of the SERVER (K^S_pub_^). The encryption guarantees the data confidentiality.
Step 6 The SERVER performs the calculation of the Diffie-Hellman values from the information coming from the CLIENT. The SERVER then performs the same actions as done by the CLIENT in step 5. It sends the resulting package to the CLIENT at the end of the process. With that, both the parties have a common key: the session key (*DH_K_*).

After exchanging the keys, the client and the server can exchange encrypted data with a symmetric key (*DH_K_*), which can last for the session.

### 4.4. Secured IoT Blockchain for Edge Computing Nodes in Layer-2

**Figure 4:**
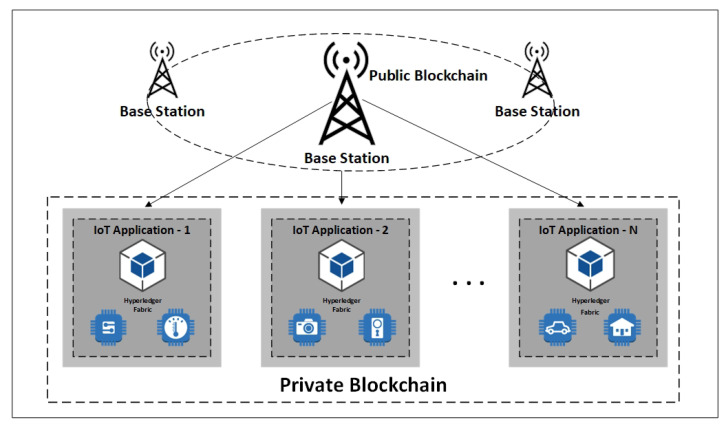
Blockchain-based edge services.

The proposed model as illustrated in Figure 4 encompasses the blockchain as part of the individual applications of the edge computing layer to provide security, data traceability, identity management, and privacy. A blockchain orchestrates a decentralized database that allows applications to trace the history of appended transactions to a shared ledger.

The main component of the proposed model in this layer is HLF blockchain framework running on the docker containers and integrated client library. The storage component is designed in a separate layer to store the actual collected data off-chain. The client library initiates the operations and communicates with other elements. The seamless provenance of metadata storage is enabled while the data checksums are recorded in a tamper-proof blockchain ledger.

#### 4.4.1. Nodes in IoT Edge Hyperledger

There are three distinct types of nodes in HLF: Peer, Orderer, and Client. The client is the node that applications use for initiating the transactions. Client nodes perform issuing transactions to the peers, collecting proposal responses, and sanding blocks for ordering. Peers are the nodes that interact with the blockchain ledger and endorse transactions through running CC. Peers are the nodes that keep the ledger in-sync across the network.

Orderers are the communication backbone for the blockchain network. They are responsible for the distribution of transactions. Furthermore, the orderer nodes are accountable for the validity and verification of responses. Moreover, the order nodes form new blocks from grouped transactions when the consensus is achieved.

**Figure 5:**
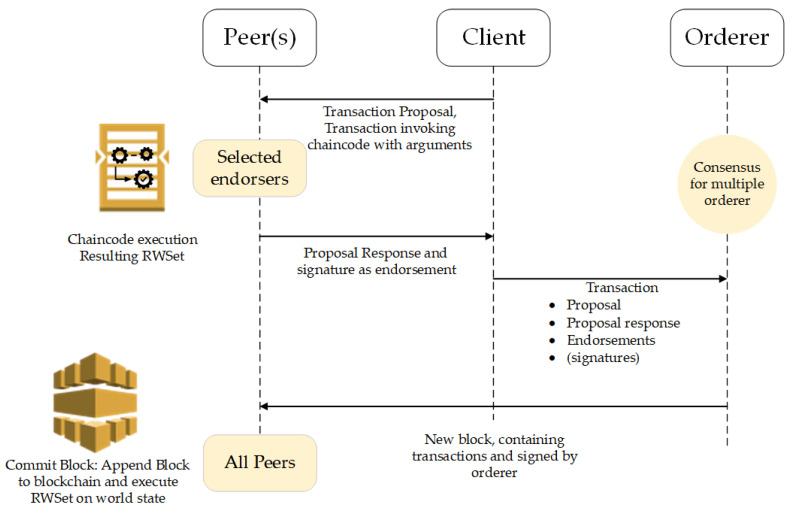
Proposed HLF network transaction flow.

Peers nodes update the ledger after the blocks are generated. Members can participate in multiple Hyperledger Blockchain networks. Transactions in each network are isolated, and this is made possible by way of what is referred to as a channel. Peers connect with the channels that can receive all the transactions that are getting broadcasted on those channels. The transaction flow is presented in Figure 5.

There are two particular types of peer nodes: Anchor and Endorser. These peers need to be configured with appropriate cryptographic materials, such as certificates. Peers in the member’s organization receive transaction invocation requests from the clients within the organization. Once transactions are created in the network and new blocks get generated, they are sent out to the peers by the ordering service. Peers receiving these blocks need to validate and update the ledger. This is managed on the peer node. Inherently, this architectural approach is highly scalable as there is no need for a centralized effort to scale the network or scale the infrastructure.

Each member organization can look at their needs and set up the needed infrastructure based on their requirements. Member organizations can have multiple peers. However, not all peers receive the block information from the Orderer—only the relevant anchor peer receives them. To avoid a single point of failure, an organization can create a cluster of the anchor peers. The anchor peers are set up and defined as part of the channel configuration. The anchor peers are by default discoverable. Peers may be marked as the endorsers or take up the endorser’s role (known as the endorsing peers). A client sends the invocation requests to the endorsing peer. On receiving the request for the invocation, the endorsing peer validates the transaction. For example, it checks whether the end-user has used a valid certificate. If the validation checks out fine, then it simulates CC.

A set of IoT edge nodes is configured to run HLF processes through Docker. Network participants run the peer process and maintain the blockchain ledger by receiving various transaction proposals. The peer process is the main component of the HLF network while hosting CC and the ledger. Network’s efficiency can be enhanced by increasing the number of running peers. However, one peer node per organization is normally sufficient. The ordering service handles blocks of ordering tasks and validates the proposed blocks by peers with a deterministic consensus algorithm. The proposed model can be enhanced through the multiple Orderers approach for fault tolerance using RAFT [12] or Kafka [76] methods.

#### 4.4.2. ChainCode in IoT Edge

Each peer participating in HLF networks keeps a copy of the ledger. The ledger consists of the blockchain and world state. Each block contains packed transactions, ordered and broadcasted by ordering service based on peer proposals. The world state database keeps the latest state in key or value form. CC is a program (smart contract) that is written to read and update the ledger state. Its operation is the process of deploying a well-developed CC onto a fabric network (channel) such that client applications can invoke CC functions. CC deployment (lifecycle ChainCode) includes: (i) install CC to selected peers, (ii) instantiate CC to a channel and specify an endorsement policy as well as initial function arguments when needed. After the deployment, invoking the ChainCode functions is accessible.

One enhancement in HLF is that the CC governance becomes decentralized. The CC package does not need to be identical across channel members. This means that organizations can extend the CC to include additional validation. Lifecycle CC includes steps in which member organizations can explicitly participate in the ChainCode deployment. The current design implements ChainCodes to manage IoT devices’ identity connected to edge gateways, store, and retrieve data from the blockchain ledger. The checksum of all collected data objects is stored in the ledger. Moreover, the location of data and the data ownership (authenticated ID) are considered to be recorded. This approach enables the system to track the data location and verify the integrity of the data. Using the certificate for invoking the transaction, the system records who and when edited or stored an item. The data lineage traceability is enabled by recording the references of the items used to generate it. The client library facilitates the ledger’s interaction to perform various functions, storing and querying the provenance information. The proposed model implements multiple endorsing nodes to ensure running the CC in a lightweight environment.

Part of the ChainCode design includes running the authentication and authorization processes for security, privacy, and identity management. Furthermore, CC tracks the owner of performed operations on data. The Client Identity (CID) CC library [58] introduced in HLF v1.1 is used in this research to save a userID issued by the Certificate Authority (CA).

#### 4.4.3. Certificate Authority

Membership Services Provider (MSP) is an abstract component of the HLF system that provides clients’ and peers’ credentials to participate in the Hyperledger Fabric network. The default MSP implementation is based on the Public-Key Infrastructure (PKI). There are two primary services provided by MSP: authentication and authorization. In PKI-based implementations, there is a need to manage the identity by way of certificates. The certificates are issued, validated, and revoked by the CA.

Each component needs to be authenticated and identified before accessing the fabric network. In a typical case, a user is issued with a digital certificate that includes proper information associated with that user. Fabric CA is the Certificate Authority developed by HLF serving a CA role. Once the Fabric CA is up and running, it can issue new certificates with the request’s specific requirement. Fabric CA can be accessed using Fabric-CA Client or Fabric SDK, both from HLF. Digital Certificate is issued by CA that is trusted by the fabric network. The user’s operation is then accepted and processed by the fabric network. The digital certificate can be issued when crypto material is generated with Cryptogen and Configtxgen binaries, or more commonly, generated through registration and enrollment on CA. The current design implements Hyperledger’s CA docker image, customized to provide persistent certificate database storage. The fabric-CA implementation has two parts: fabric-CA server and fabric-CA client. Members are issued a root certificate that they can use for issuing their own identities within their organizations. Thus, the Hyperledger fabric network can have one or more certificate authorities to manage the certificates.

#### 4.4.4. Ledger Implementation

**Figure 6:**
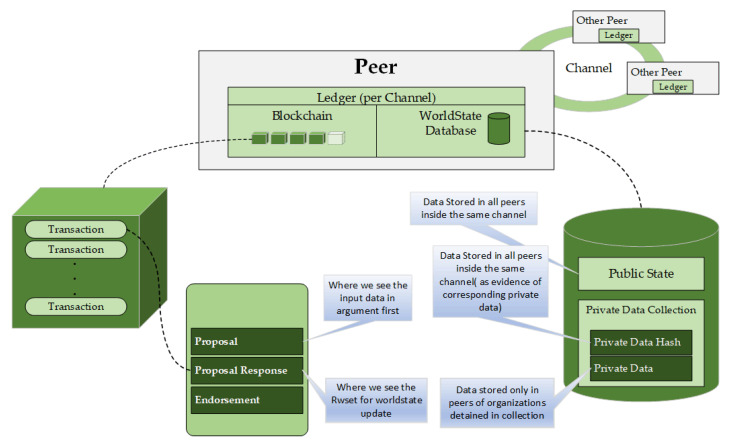
Ledger implementation flow.

HLF is a distributed ledger technology. All peers in the network have a copy replica of the ledger. The ledger has two parts: a transaction log and state database. The transaction log keeps track of all the transactions invoked against the assets. The state data are a representation of the current state of the asset at any point in time. The transaction log is implemented using the LevelDB, that is a lightweight library for building a key-value data store. It is embedded and used as part of the fabric peer implementation. Unfortunately, the LevelDB does not provide a capability for creating and executing complex queries. However, one can replace the state database (which is implemented in the LevelDB) with CouchDB that supports the creation of complex queries. Therefore, the state database is pluggable at the peer level. The transaction log is immutable. At the same time, the state data are not immutable. The creation of records in the transaction log is possible, as well as the retrieving of existing transaction records from the transaction log. However, it is not possible to update a current transaction record that is present in the log while it is possible to delete any of the transactions added to the log. From the state data perspective, create, retrieve, update, and delete operations can be carried out on the state data for an asset. The ledger implementation in the proposed model is shown in Figure 6.

### 4.5. Base Station Nodes with High Computational Power in Layer-3

BS node’s main functionality includes several tasks such as nodes management under each base station, collecting and aggregating the received data from sensing nodes, processing, analyzing, and storing the received data. As an organization manager, BS is trusted by other network participants. CH nodes (edge IoT devices) first need to be initialized and authenticated by BS before joining the network. Base stations can connect to public networks or clouds as they have robust computing and storage resources. In a public blockchain, nodes build trust in a decentralized manner through a consensus algorithm. Running public blockchain within resource constraint IoT nodes is not feasible due to the lack of needed massive capacity and time for the frequent authentication process. The unified authentication scheme is presented in Layer-2 to facilitate the joining process for nodes in a local private blockchain framework. The current hybrid design proposes a public blockchain for base stations in Layer-3 of the network model. Cluster head nodes are registered and authenticated with BS nodes through implementing the smart contracts. The node’s identity information is recorded in a public blockchain ledger.

### 4.6. Layer-4 Off-Chain Storage

Implementation of Distributed Ledgers Technologies (DLT) with blockchain is limited in terms of the amount of data stored in their ledger. The size of the shared ledger is growing incessantly, causing the system performance degradation. The solution to this challenge in the proposed design includes the use of off-chain storage. The blockchain in Layer-2 stores only the metadata’s provenance while the actual generated IoT data are stored in non-blockchain-based storage. This amount is a small fraction of the total generated data by the IoT devices. The data checksums are computed, stored, and verified with the blockchain records to ensure the integrity and immutability of the stored IoT data. The CC functions and the ledger functionality are independent of the off-chain storage choice. However, quick adding multiple storage (or other) resources is possible based on system requirements.

The current design implements SSHFS [80] as shared storage, while Raspberry Pi are employed as CHs (edge IoT devices). Thus, the choice of external shared storage needs to be aligned with the ARM64 architecture of the Raspberry Pi system. The SSHFS is a FUSE-based user-space client. It allows mounting a remote filesystem using SFTP as an underlying protocol through SSH. Most SSH servers enable and support the SFTP protocol and provide access by default. Performance evaluation of distributed storage services in the community network shows that SSHFS is comparable with other network file systems [81]. Moreover, the system enhancement is achievable with a more resilient distributed file system such as Open AFS [82] or cloud-based services such as Amazon EFS [83].

## 5. Performance Evaluation

The primary objective of any deployed blockchain applications is to maintain submitted transactions by network participants, transaction verification and ordering processes, block generation, and store the transaction outcome in a distributed ledger. Therefore, the blockchain system performance can be evaluated with the following performance metrics:

- Throughput: The maximum number of transactions that the blockchain system can handle, and record the ledger’s transaction outcomes in a given time.
- Latency: The time between the transaction invoking by a client and writing the transaction to the ledger.
- Computational Resources: Hardware and network infrastructure required for the blockchain operation.

The detailed desperation of Hyperledger performance metrics is documented in the Hyperledger Performance and Scale Working Group white paper [84].

### 5.1. Experimental Setup and Implementation

The experimental setup consists of two different environments of the same network. The first network was set up and run on virtual desktop nodes. The other system included Raspberry Pi (RPi) devices acting as IoT edge nodes. These RPis were chosen as IoT cluster heads and were connected to several small IoT sensors.

**Figure 7:**
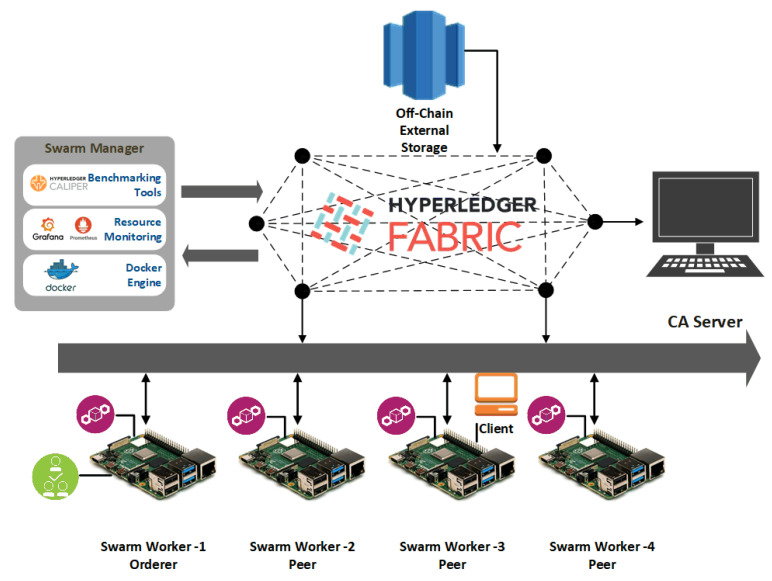
Experimental setup and system under test.

The virtual desktop setup had five virtual machines running on VMware virtual platform environment: 5 Intel(R) Xenon(R) Gold 5220 CPU@202GHz 2C2T. All nodes run Ubuntu 18.04. The official Hyperledger Fabric (version 1.4) framework was deployed as an underlying blockchain application. HLF is a permissioned open-source blockchain architecture designed for the enterprise ecosystem. Figure 7 shows the system under test high-level architecture.

The same network setup was implemented on four RPi Broadcom BCM2711 Quad-core Cortex-A72 (ARM v8) 64-bit SoC@1.5GHz devices, and one virtual desktop used as CA server. RPi nodes run the Debian 64-bit OS and nodes interconnected in a peer-to-peer network thus forming a distributed and decentralized network. Because the official HLF framework cannot be run on Raspberry Pi devices, the docker images for ARM64 architecture has been modified to support running the HLF on the RPi nodes.

Measurements on both the networks were taken enabling a comparison between the architectures. The two system setups encompass devices with dissimilar capabilities. That helped to better understand the system performance and devices’ capabilities in different scenarios of running the HLF platform. Docker containers consisted of blockchain components that were orchestrated by the Docker Swarm and deployed across the network of nodes. A client was considered to be load-generating one that could submit transactions into the system, and invoke transactions and system behaviors from it.

### 5.2. System Configurations

The system configurations encompass various tasks while taking into account also configuring system dependencies. They included Docker composes configuration, docker swarm setup, loading needed certificates and different scripts, CC configurations, external off-chain storage setting, various network access, modifying Docker images for RPi, etc. Many issues were coming from unsupported 64-bit RPi images, including software, libraries, and kernel issues. A shared Docker swarm network was implemented to manage and deploy multiple Docker containers to edge IoT nodes. Docker composes and related compose files were the central point for configuring containers deployment, modifying variables, initializing scripts, and testing the fabric network. Docker images were built to suit the RPi 64-bit ARMv8 architecture as the HLF does not officially support ARM architecture.

### 5.3. Transaction Throughput

Transaction Throughput is a performance metric defined by the Hyperledger Performance and Scale Working Group [84]. This metric represents the number of transactions processed by blockchain, leading to writing the outcome in a distributed ledger within a specific time. For this purpose and to measure the throughput, multiple rounds of benchmark applications were run on the top of the implemented HLF network with varying transaction batches. The corresponding time for each transaction and batch were measured through the benchmark application. The total time and average time were found to determine the response times and the number of transactions per minute.

#### 5.3.1. Desktop Measurements

**Figure 8:**
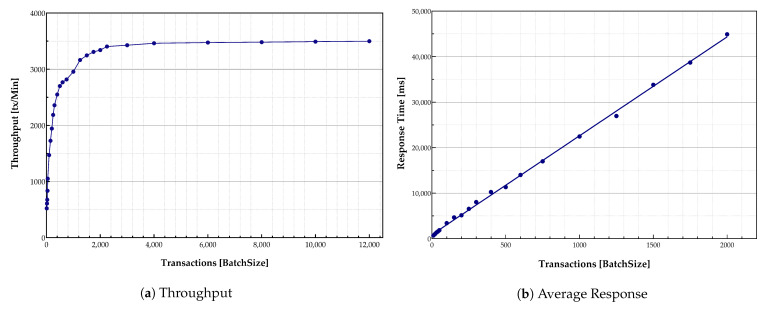
Effects of transaction sizes on the throughput and average response times in Desktop setup.

The throughput measurement was conducted by submitting several transactions together while varying load intensity levels. Figure 8a indicates exponential growth in the throughput with the batch sizes increase until it reaches its peak around 3500 transactions. Larger batch sizes can help the system to order more messages within the same block while it is submitted in the same timeout. Furthermore, Figure 8a indicates that many blocks are required to be filled up quickly to achieve higher throughput. The maximum number of transactions performed by the implemented virtual environment system was around 3500 transactions per minute, the peak system throughput. It is essential to consider that these large batch sizes were generated to evaluate the system performance. The system was limited to 58 transactions per second (approximately 3500 transactions per minute) due to the hardware capability of the virtual desktop.

Transactions response time is illustrated in Figure 8b. The response time increased with the growth in batch size. A large number of transactions caused system congestion—more transactions needed to be handled by peers and verified by the Orderer. Therefore, the individual transaction response time increased accordingly. As shown in Figure 8b, the transactions were handled quickly at the beginning of the process. However, the response time increased with the growth in the number of transactions in the queue to be handled and verified.

With the increase in the transaction arrival rate, the throughput increased linearly as expected until it flattened out at the peak point. This was because the number of ordered transactions waiting in the queue during the validation phase grew rapidly while subsequently affecting the commit latency. It shows that the validation phase was a bottleneck in the system performance. An increase in the number of Orderer nodes and validation peers could address this challenge.

#### 5.3.2. Raspberry Pi Measurements

The same system evaluation was performed in the environment consisting of RPi devices so to compare with the results obtained while using the virtual desktop setup.

**Figure 9:**
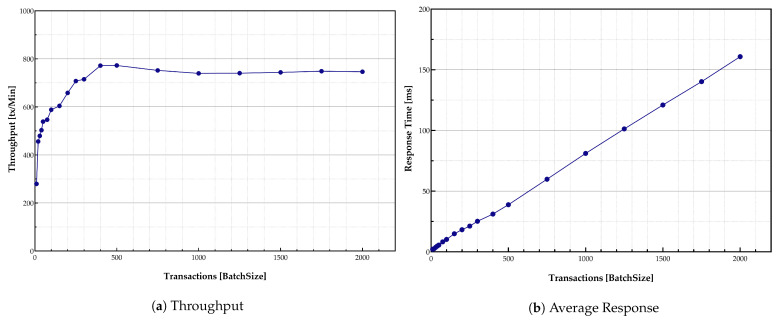
Effects of transaction sizes on the throughput and average response times in Raspberry Pi setup.

The results that are shown in Figure 9a,b confirm the same trend as was observed previously while using the desktop setup. The maximum throughput peak happened around 750 transactions batch size per minute (i.e., 12 per second), which is lower than the results for the virtual desktop case. Moreover, the higher response times than in the desktop version were observed. The peak throughput occurred in the batch sizes around 750 transactions per minute due to constraints of RPi devices in terms of the CPU capabilities.

The blockchain distributed ledger may be limited due to the amount of data stored in the blockchain system. The growth in the shared ledger causes degradation in the performance. To address this issue, the provenance of data was kept in the HLF ledger. External storage was dedicated in layer-4 of the proposed model to store the data verified by immutable blockchain records.

It should be noted that the results show satisfactory performance for the system in general. However, it is expected that the same results could be achieved by adding more clients to the system. Most of the restrictions, in this case, are related to the client’s hardware on which the applications are run and are related to the peer nodes’ limitations. The results show that storing information and recording data in the ledger do not affect the system performance any much. However, the limitations are mostly related to the time required to perform these operations as it should be done in a sequence, thereby affecting bandwidth and response times.

### 5.4. Transactions Latency

**Figure 10:**
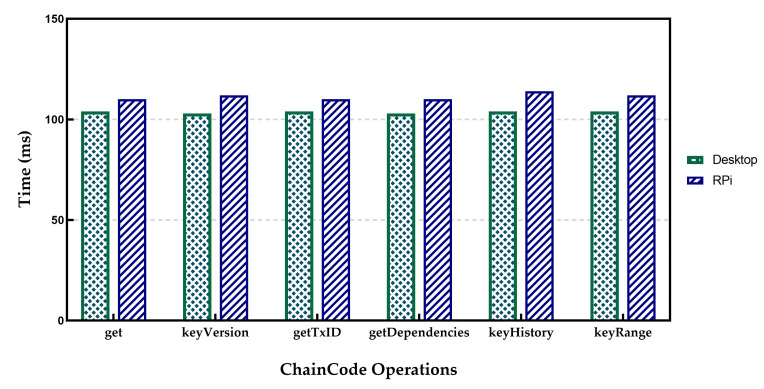
Latency for all ChainCode operation.

Transaction Latency indicates the time between the invoking of a transaction by a client and recording the transaction on the ledger. In the experimental setup, the measurements of a single transaction latency were performed by an application that sent a defined number of transactions to the HLF network while recording the individual transaction time, total average time, and corresponding statistical metrics. The results are shown in Figure 10 for CC Operation latency are the average of 100 separate operations.

Table 1 presents the results for operator SET in both desktop and RPi setup. It is evident from Table 1 that in the case of operator SET, the Raspberry Pi setup measurements were worse than those associated with the Desktop setup. The reason for this can be found in the standard deviation of related measures. The results of throughput measurements in the case of Raspberry Pi show a lot of fluctuations compared to the desktop option. It can be explained as the capability difference between the two implementations. Indeed, it took 2109 ms to submit a transaction and confirm it by running the HLF on the Desktop setup, while the time for Raspberry Pi was about 2348 ms. The Retrieving operations time for GET operators was about 100 ms in both cases. The results for RPi indicate more delays compared to the desktop environment. When the number of ordered transactions waiting in the verification process queue during the validation phase increased, it significantly increased the commit latency. Therefore, a validation phase can be considered to be a bottleneck. However, the increase in the number of involved peers also causes higher latency. Furthermore, the experiments indicate that for real applications such as IoT to achieve lower transaction latency, the use of a smaller block size with a low transaction rate would be needed. In contrast, the higher transaction rates need a larger block size to achieve higher throughput and lower transaction latency.

**Figure 11:**
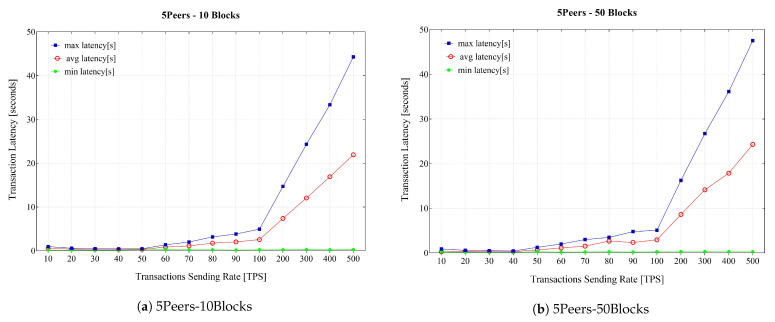
Latency vs. transaction sending rate.

The experiment was further developed with multiple rounds of the benchmark to submit transactions with different sending rates starting from 10 to 500 transactions per second (TPS) for different block sizes. The experiment aimed to measure the maximum, average, and minimum transaction latency and transaction throughput. The results are presented in Figure 11. The minimum latency remained below 1 s during the experiments, while the maximum latency proliferated as the send rate reached 100 TPS.

### 5.5. Resource Consumption

Resource measurements encompass CPU computational capability, memory, and network use. The measurements carried out with varying load levels employed edge, middle, and large load cases. The operation of storing various data sizes in the network was performed with different transactions to calculate the resource consumption. The volumes were different for desktop and Raspberry Pi network setups due to hardware limitations and RPi devices’ capability.

### 5.6. CPU and Memory Use Measurements

The CPU and memory activities were measured with the Psrecord utility [85] by attaching the processes’ pid and submitting transactions with varying data seizes. Psrecord is an open-source monitoring tool that can record real-time metrics in time-series databases. The Psrecord monitors and records a defined process. The specific usage is recorded by the Psrecord tool up to a maximum of 400% of maximum system use. The result for Orderer and ChainCode processes indicates that the resource consumption of these two processes was negligible. The Peer nodes consumed most of the memory and CPU resources. This was because the verification of the transaction and smart contracts by peer nodes required high CPU usage. Therefore, the investigation mainly dealt with the peer process and client application processes.

#### 5.6.1. Desktop Setup

**Figure 12:**
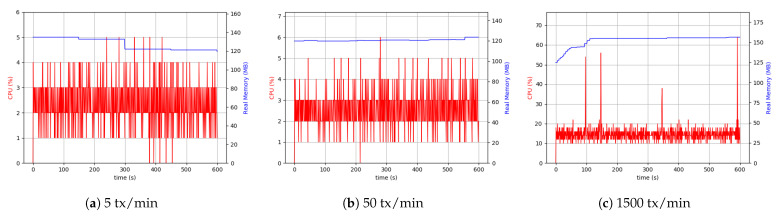
CPU and memory use for varying data sizes for peer process in the Desktop setup.

Evaluation of the CPU and memory use by the involved process provided a comprehensive view of the overhead and the impact on the device hardware. Therefore, a series of measurements were conducted to analyze resources’ consumption, including the resources of the network, CPU, and memory of the involved devices. Peer, Orderer, ChainCode, and application client processes were involved. The experiment was initiated by sending 3000 transactions per minute each of 1 KByte. The initial measurements indicated a high dependency on peer and client processes to the data sizes and throughput. However, Orderer and ChainCode processes used a small CPU capacity percentage (about 9%) and memory (approximately 16 MByte and 33 MByte). Due to that fact, the evaluation and analysis were focused more on peer and client processes’ usage of resources. With lower load sizes, the peer processes showed similar behavior. When increasing the throughput, the peer process used a higher CPU percentage (about 20%), and memory usage at around 150 MByte. The client process used approximately 40% of the CPU capacity continuously and used 120 MByte of memory. The reason for this can be attributed to multiple processes in the client. It mainly involves connecting to a peer for each transaction, invoking CC and related operators, performing related transactions, executing the proposal requests and responses related to ordered transactions. The use of resources is also increased if the client uses external storage. In this case, it needs to calculate the checksums stored in the ledger as well as storing the data in external storage. These experiments were carried out with the highest possible load amount (in the real-world scenarios, these values would be significantly lower). The results are presented in Figure 12.

Similar to the scenario with the client process, the peer process used about 40% of CPU capacity and 150 MByte of memory. One of the key elements in any HLF network is a peer node and its related processes, playing a vital role in ordering transactions. The peer node plays the role of a response coordinator to all components and from them while Peers must keep the ledger coordinated across the HLF network. Peers connect with the channels, and they can receive all the transactions that are getting broadcasted on that channel. Peer nodes’ measurements show more resource consumption than the orderer, ChainCode, and clients to synchronize with other components in the HLF network. To better evaluate and analyze peer and client processes’ behavior, the consumption of resources at different data size levels with three separate throughputs were investigated. The different levels selected were low throughput and large data size (small), low throughput and small data size (medium), and high throughput and small data size (large).

**Figure 13:**
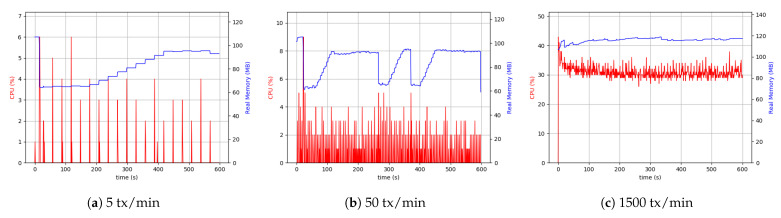
CPU and memory use for varying data sizes for client process in the Desktop setup.

The results are plotted in Figure 13 for CPU and Memory use of peer and client application processes over 10 min span with sampling per second. As seen in the plots, the peer process required a higher CPU use for the larger load with 30% increase. Similarly, the use of memory was higher, as the peer process must handle more transactions. To evaluate the client process performance and related applications, external storage was added to assess its impact on CPU and memory use. From the low number of transactions and up to many transactions, these values were sampled (Figure 13). Larger files needed more CPU and memory levels. Finally, it can be concluded that the client process can be influenced by the file size and the level of the load intensity to handle.

#### 5.6.2. Raspberry Pi Setup

Following up with analyzing the use of the resources, the RPi system setup was tested. It is crucial to acknowledge that the RPi hardware was less capable and had hardware limitations. Therefore, it was necessary to pay attention to the data sizes sent through and the number of transactions. Consequently, we considered the maximum number of transactions to be 500 per minute.

**Figure 14:**
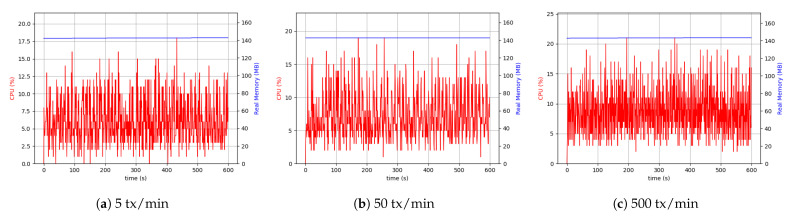
CPU and memory use for varying data sizes for peer process in RPi setup.

As is evident from Figure 14, the difference between 5 transactions per minute and 50 transactions per minute cases was more visible than the desktop setup. The continuation of the comparisons led to the conclusion that with the same throughput, the RPi uses more CPU resources (4 to 5 times more), which was interpreted as a hardware restriction inherent to RPi devices. Although it was not possible to make a comprehensive comparison between 500 transactions (tx) per minute in the case related to RPi setup and 1500 tx per minute related to desktop setup, as shown in Figure 14, the CPU usage and memory were approximately the same in both the cases.

**Figure 15:**
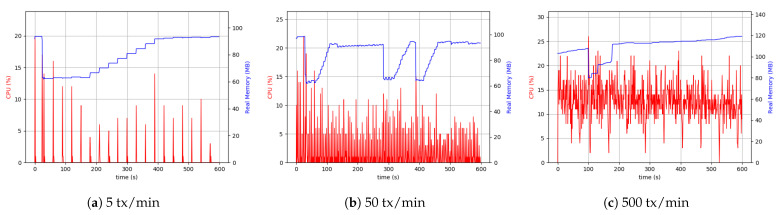
CPU and memory for varying data sizes for client process in RPi setup.

Similarly, the same measurements were performed for the client application process in the RPi setup. In this case, external data storage was considered. Figure 15 shows the results of the experiment. The higher usage of CPU was due to the difference in device-related clock rate in each of the separate setups. The peer process memory consumption was higher in the RPi setup compared to the desktop one. This can be found in peer process behavior in handling transactions. In both the setups in the client application process, the level of memory use was similar. However, in all cases, the use of 200 MByte to 300 MByte of memory was sufficient, and it was not considered the system’s main limitation. The Desktop setup’s resource consumption with a realistic transaction load size of around 50 KByte every five seconds was around 5% CPU and 15% in RPi.

### 5.7. Network Use Measurements

To assess the consumption of available network resources and to check the network overhead, launching the peer node and client application node locally could be employed to send the transactions to the orderer, other peers, and external data storage. If the peer node is launched locally, it allows us to monitor ledger updates. At the same time, all transmitted traffics between different involved participants can be checked. Furthermore, it would be possible to have an overview of all the factors of the transmitted data.

To measure and analyze network traffic, the Speedometer utility running on the Linux environment [86] was used. Speedometer measured the sent and received network traffic over a specific network interface. All other network activities were disabled. The HLF network and external storage-related communication processes were monitored using the iftop Linux monitoring tool to measure network traffic accurately. The experiments were initiated without running any processes such as the Docker, and only the process run by the operating system to be monitored was allowed. The results show that baseline 3–5 KByte/s data can be written off to others as the network traffic.

**Figure 16:**
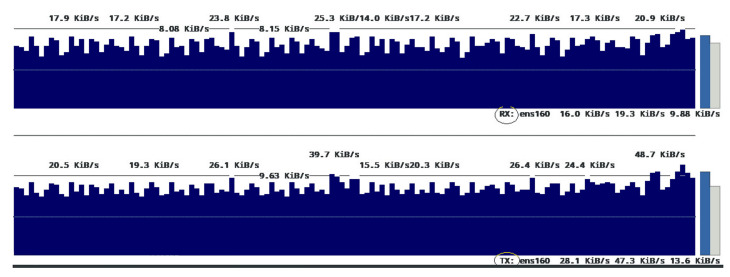
Network use for peer process with no transactions.

**Figure 17:**
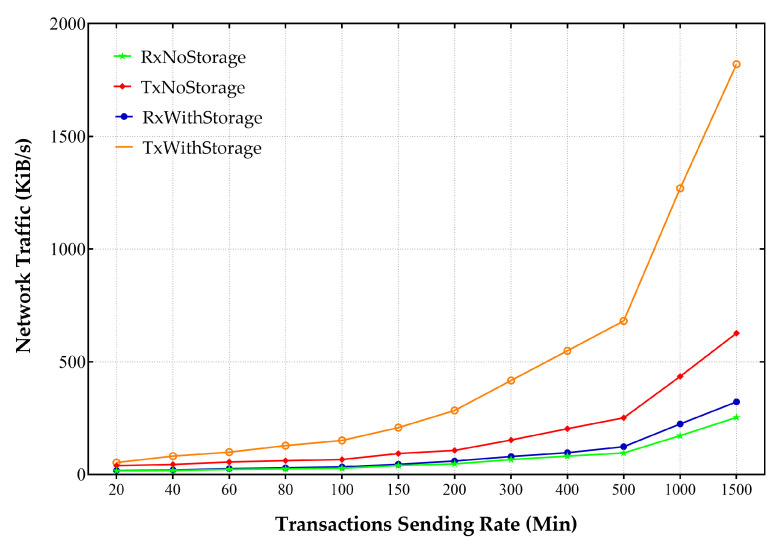
Network use vs. load sizes with/without external storage.

With running the HLF, significant changes in network traffic were detectable. Figure 16 displays that with the onset of the peer process, network traffic increased by about five times compared to the baseline mode. In this case, there were no transactions between peers. The main reason for this was the beginning of the communication between peer process and network components, to have ledger consistency and reaching a synchronization through the gossip protocol. For further analysis and finding out how network resources would be affected by offered load, different offered load levels were engaged, and various modes were evaluated with and without external storage resources. The relevant results are presented in Figure 17.

The results show that receiving and sending traffic to perform transactions every 5 s occupies something about 1–40 KByte/s spectrum. Involving an external storage source significantly increases traffic and increases its range to about 100 KByte/s. This increase was also visible in the incoming traffic and indicated by the file storage’s confirmation in the shared folder. Further increase in the number of transactions would increase the sent and received traffic.

## 6. Conclusions

Providing security to massive interconnected IoT devices while ensuring the scalability of IoT systems with minimum resource requirements is a challenging problem. Additionally, the heterogeneity and diversity of connected devices within the IoT realm make it even more challenging. Therefore, the interoperability, identity, and privacy of IoT systems need to be guaranteed securely. The existing centralized solutions, such as a cloud-centric model, are costly. Moreover, these solutions’ latency is also noticeable. Furthermore, the single point of failure issue is a considerable risk to the security of the centralized solutions. Blockchain technology is a promising solution to provide security for IoT devices while leveraging trust and interoperability.

This paper presented an implementation of the Hyperledger Fabric Blockchain platform as a permissioned blockchain technology integrated with edge IoTs to test and analyze the performance of the proposed BlockChain-based multi-layer IoT security model. The presented proof of concept was implemented using two different environment setups on the Raspberry Pi devices and VMware Virtual desktops. The performance metrics such as transaction throughput, transaction latency, computational resources, and network use of the implemented networks, were evaluated. The implemented prototype facilitates the record of sensing data by IoT devices (metadata) in a tamper-proof and transparent blockchain-based framework to provide data traceability. Moreover, the framework’s security is guaranteed by implementing a layer-wise blockchain approach and local authentication process for IoT nodes in each cluster. The client application is developed with the help of Hyperledger Node SDK where various Hyperledger ChainCodes help to perform local authentication and authorization. Moreover, they facilitate the record of file pointers to provide checksums traceability and data validation.

The presented findings indicate a significantly optimal throughput for IoT applications. Peers and clients’ processes are the primary source of resource consumption in the network. The Orderer and ChainCode use fewer resources compared to the peer process. Experimental results show a significant increase in throughput of approximately six times compared to the optimal scale implementation of HLF. The Desktop setup’s resource consumption with a realistic transaction load size of around 50 KByte every five seconds is around 5% CPU and for the RPi setup is around 15% CPU. Peer and client processes are the primary resource consumers in HLF as our measurements indicate an average of 40% to 50% CPU consumption respectively at full load, while these measurements for the Orderer process and ChainCode use an average of about 10% of CPU resources. The deployed model could retrieve a single record in 100 ms. However, the use of the built-in ChainCode queries allows retrieving 10 dependent IoT records in 102 ms. The empirical results all indicate low overhead for running the proposed model.

Further work will consider the deployment of the proposed model in larger-scale IoT scenarios significantly increasing the number of peers for the empirical analysis of the system performance for both overall and detailed Fabric performance metrics, including throughput, latency, block size, endorsement policy, and scalability.

## Figures and Tables

**Table 1:** Statistics analysis of SET ChainCode latency.

Setup Environment	Avg	Std	Med	Max	Min
Desktop	2109	42.5	2105	2518	2103
RPi	2348	252	2306	4029	2204

## Data Availability

The data presented in this study are available on request from the corresponding author.

## Abbreviations

The following abbreviations are used in this manuscript:

BFT	Byzantine Fault Tolerant
BS	Base Station
CA	Certification Authority
CC	ChainCodes
CH	Cluster Head
CID	Client Identity
CRL	Certificate Revocation List
CPSs	Cyber-Physical Systems
dApps	distributed Applications
DDoS	Distributed Denial-of-Service
DHE	Diffie-Hellman Ephemeral
DLTS	Distributed Ledger Technologies
DPoS	Delegated Proof of Stake
ECC	Elliptic Curve Cryptography
HLF	Hyperledger Fabric
IoT	Internet of Things
IIoT	Industrial IoT
MAC	Message Authentication Code
MSP	Membership Services Provider
NFS	Network File Systems
OPM	Open Provenance Model
PBFT	Practical Byzantine Fault Tolerance
PoA	Proof of Authority
PoB	Proof of Bandwidth
PoET	Proof of Elapsed Time
PoS	Proof of Stake
PoW	Proof of Work
RPi	Raspberry Pi
RSA	Rivest–Shamir–Adleman
SDN	Software Defined Networking
SSL	Secure Sockets Layer
TSL	Transport Layer Security
WSNs	Wireless Sensor Networks
